# Effects of Genetic Polymorphism in CYP2D6, CYP2C19, and the Organic Cation Transporter OCT1 on Amitriptyline Pharmacokinetics in Healthy Volunteers and Depressive Disorder Patients

**DOI:** 10.3389/fphar.2021.688950

**Published:** 2021-05-21

**Authors:** Johannes Matthaei, Jürgen Brockmöller, Werner Steimer, Konstanze Pischa, Stefan Leucht, Maria Kullmann, Ole Jensen, Typhaine Ouethy, Mladen Vassilev Tzvetkov, Muhammad Rafehi

**Affiliations:** ^1^ Institute of Clinical Pharmacology, University Medical Center Göttingen, Georg-August University, Göttingen, Germany; ^2^ Institute for Clinical Chemistry and Pathobiochemistry, Klinikum rechts der Isar, Technical University of Munich, Munich, Germany; ^3^ Section Evidence Based Medicine in Psychiatry and Psychotherapy, Department of Psychiatry and Psychotherapy, Klinikum rechts der Isar, Technical University of Munich, Munich, Germany; ^4^ Institute of Pharmacology, Center of Drug Absorption and Transport (C_DAT), University Medicine Greifswald, Greifswald, Germany

**Keywords:** amitriptyline, CYP2C19, CYP2D6, drug transport, nortriptyline, OCT1, organic cation transporter 1, SLC22A1

## Abstract

The tricyclic antidepressant amitriptyline is frequently prescribed but its use is limited by its narrow therapeutic range and large variation in pharmacokinetics. Apart from interindividual differences in the activity of the metabolising enzymes cytochrome P450 (CYP) 2D6 and 2C19, genetic polymorphism of the hepatic influx transporter organic cation transporter 1 (OCT1) could be contributing to interindividual variation in pharmacokinetics. Here, the impact of OCT1 genetic variation on the pharmacokinetics of amitriptyline and its active metabolite nortriptyline was studied *in vitro* as well as in healthy volunteers and in depressive disorder patients. Amitriptyline and nortriptyline were found to inhibit OCT1 in recombinant cells with IC_50_ values of 28.6 and 40.4 µM. Thirty other antidepressant and neuroleptic drugs were also found to be moderate to strong OCT1 inhibitors with IC_50_ values in the micromolar range. However, in 35 healthy volunteers, preselected for their OCT1 genotypes, who received a single dose of 25 mg amitriptyline, no significant effects on amitriptyline and nortriptyline pharmacokinetics could be attributed to OCT1 genetic polymorphism. In contrast, the strong impact of the CYP2D6 genotype on amitriptyline and nortriptyline pharmacokinetics and of the CYP2C19 genotype on nortriptyline was confirmed. In addition, acylcarnitine derivatives were measured as endogenous biomarkers for OCT1 activity. The mean plasma concentrations of isobutyrylcarnitine and 2-methylbutyrylcarnitine were higher in participants with two active OCT1 alleles compared to those with zero OCT1 activity, further supporting their role as endogenous *in vivo* biomarkers for OCT1 activity. A moderate reduction in plasma isobutyrylcarnitine concentrations occurred at the time points at which amitriptyline plasma concentrations were the highest. In a second, independent study sample of 50 patients who underwent amitriptyline therapy of 75 mg twice daily, a significant trend of increasing amitriptyline plasma concentrations with decreasing OCT1 activity was observed (*p* = 0.018), while nortriptyline plasma concentrations were unaffected by the OCT1 genotype. Altogether, this comprehensive study showed that OCT1 activity does not appear to be a major factor determining amitriptyline and nortriptyline pharmacokinetics and that hepatic uptake occurs mainly through other mechanisms.

## Introduction

Amitriptyline (AT) is a tricyclic antidepressant that has been in use for the therapy of major depression and other psychiatric disorders since the 1960ies. It is still frequently used today but usually as second-line therapy, due to its risk for severe adverse reactions. In addition, AT and its active metabolite nortriptyline (NT) show large interindividual variation in pharmacokinetics and, accordingly, there is substantial interest in therapy individualisation by drug monitoring and using molecular genetic biomarkers for polymorphic drug membrane transport and biotransformation (Hiemke et al., 2018). The mechanism of action involves reuptake inhibition of serotonin and noradrenaline in the synaptic cleft (Gillman, 2007). Adverse reactions are concentration-dependent and can result from antagonism of H1 histamine, alpha-1-adrenergic, and muscarinic receptors (Richelson, 1979; Kachur et al., 1988; Goldman et al., 1989; Ramakrishna and Subhash, 2012). Apart from being an antidepressant, AT is also used at lower doses for migraine prophylaxis, the management of neuropathic pain, in irritable bowel syndrome, and for the treatment of fibromyalgia (Moore et al., 2015; Rico-Villademoros et al., 2015; Silberstein, 2015; Schneider et al., 2019).

**FIGURE 1:**
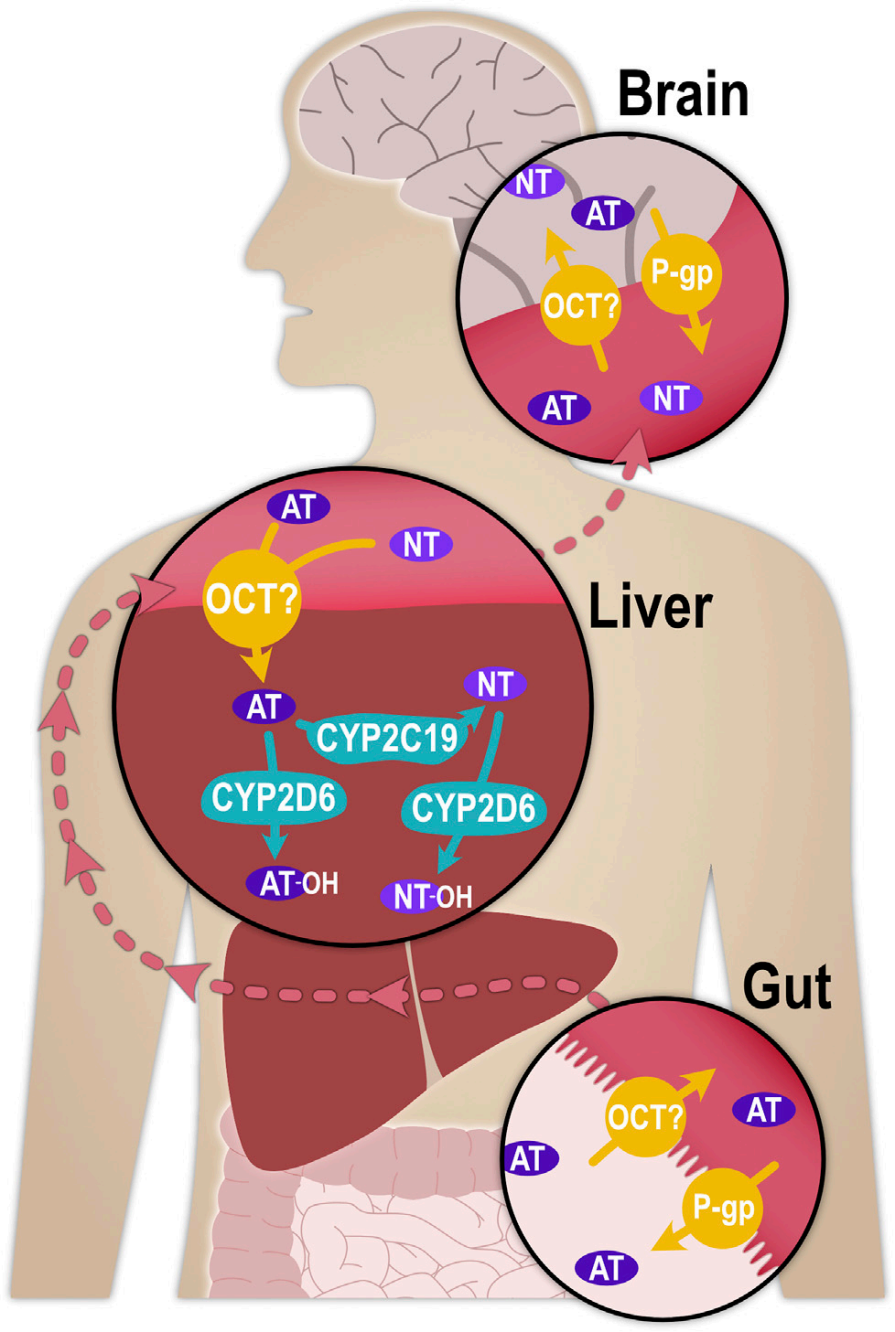
Schematic illustration of the processes that determine the pharmacokinetics of AT and NT. P-glycoprotein (P-gp, MDR1, ABCB1), as part of the blood-brain-barrier, was shown in mice to transport AT and NT from the central nervous system into brain capillaries, thereby determining their concentrations at the synapse (Uhr, 2000; Grauer and Uhr, 2004; Uhr et al., 2007).

Upon systemic absorption, AT is subject to extensive hepatic metabolism, with less than 5% excreted unchanged in urine (Rudorfer and Potter, 1999). AT is metabolised mainly by cytochrome P450 (CYP) 2D6 into hydroxylated metabolites and by CYP2C19 to NT (Figure 1), which itself is also a tricyclic antidepressant (Breyer-Pfaff, 2004; Hicks et al., 2017). In fact, both, the more serotoninergic AT and its more noradrenergic metabolite NT, contribute to the therapeutic effects after AT administration (Hiemke et al., 2018). Both CYP2D6 and CYP2C19 are genetically highly polymorphic (Dalén et al., 1998; Zhou, 2009; Bahar et al., 2017; Sienkiewicz-Oleszkiewicz and Wiela-Hojeńska, 2018), and the substantial impact this has on AT and NT pharmacokinetics has been known for several decades (Mellström et al., 1983; Baumann et al., 1986; Steimer et al., 2004; Steimer et al., 2005; Milosavljevic et al., 2021). It has since been strongly recommended to implement regular CYP2D6 and CYP2C19 genotyping in AT therapy and consider personalised dose adjustments (Kirchheiner et al., 2001; Hicks et al., 2017).

For hepatic metabolism to occur, AT must first enter the hepatocytes. With a pK_a_ of 9.4, approximately 99% of AT is positively charged at physiological pH and, despite its lipophilicity (logD_7.4_ of 3), it may traverse cell membranes more efficiently by carrier-mediated transport than by non-ionic diffusion. One possible influx transporter with particular relevance for hepatic uptake could be the organic cation transporter 1 (OCT1; *SLC22A1*), a member of the Solute Carrier (SLC) family with a very broad substrate profile (Koepsell, 2020). OCT1 is abundantly expressed at the sinusoidal membrane of hepatocytes (Nishimura and Naito, 2005), where it mediates the hepatic uptake of organic, mostly cationic endogenous and exogenous small molecule compounds. A large number of inherited variants in the gene coding for OCT1 with comparatively high population frequencies have been described, and carriers of some of these variants showed greatly reduced or completely deficient transport activity (Seitz et al., 2015). OCT1 polymorphism may thus partially account for interindividual differences in the pharmacokinetics of numerous drugs (Tzvetkov et al., 2011; Tzvetkov et al., 2013; Tzvetkov et al., 2018; Matthaei et al., 2019; Koepsell, 2020; Jensen et al., 2021). The increased plasma concentrations of these drugs in some patients as a result of OCT1 (partial or complete) deficiency may lead to more severe adverse reactions. This could potentially be the case for AT and NT as well, and the aim of this study was to explore this possibility.

Genome-wide association studies have found a strong association between the *SLC22A1* locus and plasma concentrations of acylcarnitines, which are intermediate metabolites of mitochondrial oxidation reactions (Suhre et al., 2011). This provides further insights into the potential physiological functions of OCT1. It is also of medical relevance, as plasma acylcarnitine concentrations have been associated with metabolic disorders, including obesity and diabetes (Adams et al., 2009; Mihalik et al., 2010; Mai et al., 2013). Isobutyrylcarnitine (IBC) has been proposed to function as an endogenous biomarker for studying OCT1 *in vivo* (Luo et al., 2020). Thus, the effects of AT on plasma IBC concentrations were studied here as well.

The purpose of this study was to investigate whether OCT1 polymorphism may determine the pharmacokinetics of AT and its clinically relevant metabolite NT. This was studied here *in vitro*, in healthy volunteers, and in depressive disorder patients. In addition, the impact of CYP2D6 and CYP2C19 genetic polymorphism on AT and NT pharmacokinetics was characterized further and possible effects of AT on plasma IBC concentrations were explored.

## Materials and Methods

### *In vitro* OCT1 Inhibition Experiments

The inhibition of OCT1 by different psychotropic drugs was studied in transport experiments using HEK293 cells stably transfected with wild-type OCT1. The cells were generated by targeted chromosomal integration using the Flp-In system (Thermo Fisher Scientific, Darmstadt, Germany), as has been described in detail before (Saadatmand et al., 2012). The cells were cultured in Dulbecco’s Modified Eagle’s Medium supplemented with 10% foetal bovine serum, 100 U/ml penicillin, and 100 μg/ml streptomycin at 37°C, 5% CO_2_, and 95% relative humidity. Cells were kept in culture for no more than 30 passages. All buffers and reagents were purchased from Thermo Fisher Scientific (Darmstadt, Germany).

Approximately 48 h before the transport experiments, recombinant OCT1-expressing cells and empty vector-transfected control cells were seeded at a density of 4 × 10^5^ cells/well in 12-well plates coated with poly-D-lysine and incubated as described above. On the day of the experiment, the cells were washed twice with prewarmed (37°C) Hank’s Balanced Salt Solution (HBSS). They were subsequently incubated for 3 min at 37°C with 1 µM of the fluorescent OCT1 substrate ASP^+^ (4-(4-(dimethylamino)styryl)-*N*-methylpyridinium iodide) and increasing concentrations of the antidepressants in 500 µl HBSS. The reaction was stopped by adding 2 ml ice-cold HBSS. This was subsequently removed and the cells were lyzed in 500 µl radioimmunoprecipitation assay (RIPA) buffer for 10 min under shaking. The cell uptake of ASP^+^ was quantified using a Tecan Ultra microplate reader (Tecan Group AG, Männedorf, Switzerland) at excitation wavelength 485 nm and emission wavelength 612 nm. The intracellular ASP^+^ concentrations were normalised to the total amount of protein in the sample that was determined using the bicinchoninic acid assay (Smith et al., 1985). IC_50_ values were calculated using SigmaPlot 11 (Systat Software GmbH, Erkrath, Germany) and Prism 5 (GraphPad Software, San Diego, CA, United States).

### Subjects and Study Designs

#### Study in Healthy Volunteers

In this open-label study, the pharmacokinetics of 25 mg AT were analysed in relation to OCT1, CYP2D6, and CYP2C19 genotypes. In total, 35 unrelated healthy volunteers participated in this study. When considering the frequent OCT1 alleles *2, *3, *4, *5, and *6 to be functionally deficient, approximately 25% of Europeans are carriers of at least one deficient OCT1 variant and about 7% are homozygously deficient with respect to OCT1 (Seitz et al., 2015). In order to enrich the study sample with the less frequent functionally deficient OCT1 variants, participants were selected based on OCT1 genotype from an internal database at the Institute of Clinical Pharmacology of the University Medical Center Göttingen. All volunteers who are listed in the internal database had agreed to it and the database was approved by the ethics committee of the University of Göttingen. The number of participants for each group (carriers of 2, 1, and 0 active OCT1 alleles; Tables 1, 2) was calculated to achieve 80% power to identify a 50% difference in the area under the plasma concentration-time curve (AUC; the primary parameter in this study) in the carriers of 2 compared to the carriers of 0 active OCT1 alleles with a type-I (alpha) error of 5% and assuming a 35% standard deviation of the AUC in both groups. A 50% decrease in clearance is a reasonable effect size in comparison with known effects of CYP2D6 polymorphism on the AUCs of AT and NT (Kirchheiner et al., 2004) and considering that clinical drug dose adjustments are typically by about 50% or more. Additional subjects with heterozygous genotypes were included to provide a better understanding of the effects of specific variants and the mode of inheritance. All volunteers gave their written informed consent before participation in the study. The study was approved by the ethics committee of the University of Göttingen and the German Federal Institute for Drugs and Medical Devices (BfArM). It was registered at the clinical trials databases Clinicaltrials.gov (NCT02054299) and EudraCT (number 2012-003546-33).

**TABLE 1:** Demographic data of the healthy volunteer study population stratified by OCT1 genotype.

Parameter	2 active OCT1 alleles (*n* = 14)	1 active OCT1 allele (*n* = 9)	0 active OCT1 alleles (*n* = 12)	Total study population (*n* = 35)
Mean age (years)	25	27	29	27
Sex	7 (50%) male	4 (44%) male	5 (42%) male	16 (46%) male
Mean body height (cm)	177	178	172	175
Mean body weight (kg)	71	72	70	71
Mean body mass index	23	23	24	23
Ethnicity	All caucasian	All caucasian	All caucasian	All caucasian
Smoking habit	1 smoker	0 smokers	1 smoker	2 smokers

Healthy male and female volunteers aged between 18 and 50 y with a body mass of at least 48 kg and a body mass index of 17–32 were eligible for inclusion. Volunteers who underwent regular drug treatments other than oral contraceptives or who suffered from any relevant chronic illness, as well as pregnant or lactating women, were not included. All subjects were healthy according to a detailed medical history, medical examination, electrocardiogram, urine status and clinical chemistry, and haematology parameters (sodium, potassium, total bilirubin, aspartate aminotransferase, alanine-aminotransferase, creatinine, C-reactive protein, thyroid-stimulating hormone, haemoglobin, erythrocyte, thrombocyte, and leucocyte counts).

After overnight fasting, a single dose of 25 mg AT (Amitriptylin-dura^®^, Mylan dura GmbH, Darmstadt, Germany) was orally administered to each subject. Blood samples were taken before AT administration and at 1, 2, 4, 6, 8, 12, 24, and 48 h after administration. The blood samples were centrifuged at room temperature for 10 min and the plasma was stored at −20°C before the concentration analyses. Blood pressure and heart rate were measured, a resting electrocardiogram was taken, pupillometry measurement was taken, and the participants were asked to report on any adverse events and possible symptoms (specifically, we asked for possible headache, fatigue, sleepiness, visual or hearing impairments, restlessness, nausea, dizziness, dry mouth, tremor, and a sensation of cold) using visual analogue scales 1 h before AT administration and at the following time points after administration: 65 min, 3, 5, 7, 11.5, 23.5, and 47.5 h.

#### Study in Depressive Disorder Patients

In addition to the study in healthy volunteers described above, possible effects of the OCT1 genotype on AT pharmacokinetics were also investigated in 50 patients suffering from at least medium-grade depressive disorder. These patients had been recruited within a previous study, in which the effects of different CYP2D6 and CYP2C19 genotypes on AT and NT pharmacokinetics as well as on adverse effects and therapy response were investigated. A detailed description of the study sample, the study design, and the results pertaining to CYP2D6 and CYP2C19 polymorphism are found in the respective publications (Steimer et al., 2004, 2005). Briefly, 75 mg AT was administered twice daily at 12 h dosing intervals. Any drugs or dietary ingredients that might interfere with CYP2D6 or CYP2C19 metabolism were avoided whenever possible. Blood samples (12-hour-trough levels) were taken on days 0, 7, 14, 18, and 21, centrifuged, and stored at 4°C for genotyping (CYP2D6 and CYP2C19) and concentration analyses. The blood samples were subsequently stored at −20°C and later genotyped for OCT1. The study has been approved by the ethics committee of the Technical University of Munich, Germany.

### Bioanalytics

#### Study in Healthy Volunteers

The peripheral venous blood samples of the healthy volunteers were treated with ethylenediaminetetraacetic acid (EDTA) for anticoagulation, centrifuged within 30 min after withdrawal (3,100 × *g*, 10 min, room temperature), and the plasma was stored at −20°C. For determining the plasma concentrations of AT, NT, IBC, 2-methylbutyrylcarnitine, and propionylcarnitine, plasma samples were mixed with twice the volume precipitation reagent of 10% (v/v) methanol and 90% (v/v) acetonitrile that included the corresponding deuterated internal standards AT-d6 (Biozol Diagnostica GmbH, Eching, Germany), NT-d3, IBC-d6, 2-methylbutyrylcarnitine-d9, and propionylcarnitine-d3 and d9 (all Santa Cruz Biotechnology, Heidelberg, Germany) and shaken for 15 min. After centrifugation (13,000 rpm, 15 min, room temperature), two-thirds of the supernatant were transferred to a new reaction tube and evaporated at 40°C under nitrogen flow. The residue was reconstituted under shaking in 0.1% methanoic acid and briefly centrifuged before quantification using a Nexera UHPLC system (Shimadzu, Kyoto, Japan) coupled to an API 4000 tandem mass spectrometer (AB Sciex, Darmstadt, Germany). Separation was done using a Brownlee SPP RP-Amide column (4.6 × 100 mm inner dimensions, 2.7 µm particle size; PerkinElmer, Rodgau, Germany) with a Phenomenex C18 pre-column (4 × 2 mm, Phenomenex, Aschaffenburg, Germany). For AT and NT, the mobile phase consisted of 0.1% (v/v) methanoic acid, 5.3% (v/v) methanol, and 31.7% (v/v) acetonitrile in water. For the carnitine derivatives, it consisted of 0.1% (v/v) methanoic acid, 0.43% (v/v) methanol, and 2.57% (v/v) acetonitrile in water. The lower limit of quantification was 0.5 ng/ml for AT and 0.1 ng/ml for NT. Precision and accuracy were controlled by additional control samples spiked with 2 and 20 ng/ml of AT and NT, resulting in coefficients of variation of 6.0 and 3.8% (means of 2.02 and 19.5 ng/ml) for AT and of 3.8 and 2.7% (means of 1.91 and 18.8 ng/ml) for NT. The mass spectrometry detection parameters are listed in Supplementary Table S1.

#### Study in Depressive Disorder Patients

The serum concentrations in depressive disorder patients were determined either by the Emit^®^ immunoassay specific for AT and NT or a commercial high-performance liquid chromatography assay (Bio-Rad Laboratories GmbH, Feldkirchen, Germany), as described before (Steimer et al., 2004; Steimer et al., 2005).

### Genotyping

For both studies, genomic DNA was isolated from venous blood samples via automated solid phase extraction (EZ1 DNA Blood kit; Qiagen, Hilden, Germany). The following genetic variants were analysed using single-base primer extension using fluorescence-labelled dideoxynucleotides (described by Seitz et al. (2015) and Kirchheiner et al. (2008)) for OCT1: *1 (wild-type), *2 (M420del, rs72552763), *3 (R61C, rs12208357), *4 (G401S, rs34130495), *5 (G465R, rs34059508 in combination with M420del, rs72552763), *6 (C88R, rs55918055 in combination with M420del, rs72552763), *7 (S14F, rs34447885), *9 (P117L, rs200684404), and *10 (S189L, rs34104736); for CYP2D6: *1, *2, *3, *4, *5, *6, *9, *10, *35, *41, and gene duplication. The CYP2C19 variants *2 (rs4244285) and *17 (rs12248560) and the OCT1 variant *8 (Arg488Met, rs35270274) were genotyped using a TaqMan SNP genotyping assay (Life Technologies). Almost all samples were genotyped in duplicate, with 100% concordant results.

### Statistics

For the study in healthy volunteers, the primary endpoints were the AUCs of plasma AT and NT concentrations. Secondary endpoints were the other pharmacokinetic parameters of AT and NT, as well as heart rate, blood pressure, pupil size effects, and possible adverse events (headache, fatigue, visual or hearing impairments, restlessness, nausea, dizziness, dry mouth, tremor, as well as sensations of hypothermia and heart palpitations determined using a visual analogue scale test). Pharmacokinetic parameters were calculated by non-compartmental analysis using Phoenix 64 WinNonlin version 6.3 (Certara Inc., Princeton, NJ, United States). AUC_inf_ of AT was calculated from the time of dosing using the linear/log trapezoidal rule and extrapolated to infinity based on the last predicted concentration and using the terminal elimination rate constant (lambda z). AUC of NT was calculated from the time of dosing until the last measurement at 48 h using the linear/log trapezoidal rule, as a decline in NT concentrations was not observed in some subjects and extrapolation to infinity thus not possible. Further parameters that were studied included the total plasma clearance after oral administration (CL/F) and the terminal half-life (t_1/2_), which were calculated as CL/F = dose/AUC_inf_ and t_1/2_ = ln (2)/lambda z.

The correlation between AT and NT plasma AUC (study in healthy volunteers) or mean plasma concentration per dose unit (study in depressive disorder patients) and OCT1, CYP2D6, and CYP2C19 genotypes were calculated using the Jonckheere-Terpstra non-parametric analysis, which takes into consideration the *a priori* ordering (or trend) in gene activities from zero to normal to ultra-rapid (for CYP2C19 and CYP2D6). To do so, the genotypes were categorized into 0, 1, or 2 active alleles for OCT1, into 0, 0.5, 1, 1.5, 2, 2.5, or 3 active alleles for CYP2D6, and into 0, 1, 1.5, 2, 2.5, or 3 active alleles for CYP2C19, depending on their known effects on transporter/enzyme activity. OCT1 alleles *2, *3, *4, *5 were classified as being zero active. However, given the substrate-dependent effects of OCT1*2, calculations were repeated with OCT1*2 classified as being fully active. A semi-quantitative gene dosage was calculated for CYP2D6, as has been described earlier (Steimer et al., 2004). For calculating a CYP2C19 activity score, CYP2C19*2 was regarded as zero active, CYP2C19*1 was classified as 1, and CYP2C19*17 as 1.5. Additional multiple linear regression analyses included sex, age, body mass index, and glomerular filtration rate.

### Dose-Adjustment Calculations

The genotype-based dosage adjustment recommendations were calculated by using the equations described in Stingl et al. (2013) (supplementary data), modified to base these calculations on AUC data instead of clearance values. The adjusted dose was thereby calculated for CYP2D6 extensive metabolisers (EM) as D_EM_ = 100/(0.1 × AUC_EM_/AUC_PM_ + 0.4 × AUC_EM_/AUC_IM_ + 0.5) and for CYP2C19 EM as D_EM_ = 100/(0.03 × AUC_EM_/AUC_PM_ + 0.27 x AUC_EM_/AUC_IM_ + 0.7). The dose adjustments for the poor (PM), intermediate (IM), and ultra-rapid (UM) metaboliser phenotypes were calculated as follows: D_PM or IM or UM_ = D_EM_ x AUC_EM_/AUC_PM or IM or UM_. The multipliers in the EM calculations account for the typical population frequencies of the respective genotypes in European populations (e.g. 0.1 for 10% of CYP2D6 PM). The rationale behind these calculations is that the average recommended drug dose usually determined without considering the genotypes was chosen as the average optimum for populations with the given genotype frequencies (Kirchheiner et al., 2001).

## Results

### *In vitro* Inhibition of OCT1 by Different Psychotropic Drugs

**FIGURE 2:**
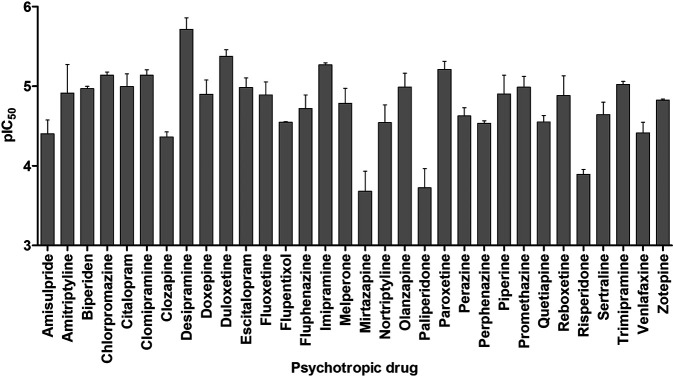
*In vitro* assessment of OCT1 inhibition by a range of different antidepressant and neuroleptic drugs. Shown is the mean negative logarithm of the IC_50_ values of 3–4 independent experiments, the error bars indicate the SEM.

Thirty-two clinically relevant antidepressants, neuroleptics, and an anticholinergic drug for the treatment of Parkinson’s disease were screened for OCT1 inhibition. These have been selected based on their positive charge at physiological pH, as charged compounds are mostly unable to efficiently traverse biological membranes through passive diffusion and their pharmacokinetics might depend on transport proteins, such as OCT1. An inhibitor for a transporter does not necessarily have to be a substrate as well, but for many compounds, this is indeed the case. The psychotropic drugs were assessed for their potential to inhibit cell uptake of the fluorescent OCT1 model substrate ASP^+^ (4-(4-(dimethylamino)styryl)-*N*-methylpyridinium iodide) in OCT1-overexpressing cells. As shown in Figure 2, most of the tested compounds showed inhibitory potencies in the low to mid-micromolar range, indicating that these are moderate to strong inhibitors of OCT1. AT showed a mean IC_50_ value of 28.6 ± 18.9 µM and NT of 40.4 ± 16.2 µM, which is in agreement with previously reported data (Haenisch et al., 2012; Zhu et al., 2018). Because of this interaction with OCT1 and its widespread clinical use, AT was further analyzed with respect to the impact of genetic variation in OCT1 in volunteer and patient studies.

### Pharmacokinetics of Amitriptyline and Nortriptyline in Relation to OCT1, CYP2D6, and CYP2C19 Genotypes in Healthy Volunteers

In the clinical study in healthy volunteers, preselected according to their OCT1 genotype, 35 volunteers (19 female and 16 male) received 25 mg of AT as a single dose. The study participants were between 18 and 48 y of age, with a mean age of 27 y. The mean body mass index was 23.0 kg/m^2^. Stratified by OCT1 genotype, 14 subjects were homozygous carriers of the active OCT1*1 (wild-type) allele, nine subjects carried one active allele (OCT1*1) and one allele with no or reduced activity (*2,*3, *4), and 12 subjects were carriers of two OCT1 alleles with no or reduced activity (*2, *3, *4, *5). There were no significant differences in demographic data between the OCT1 genotypes (Table 1).

**FIGURE 3:**
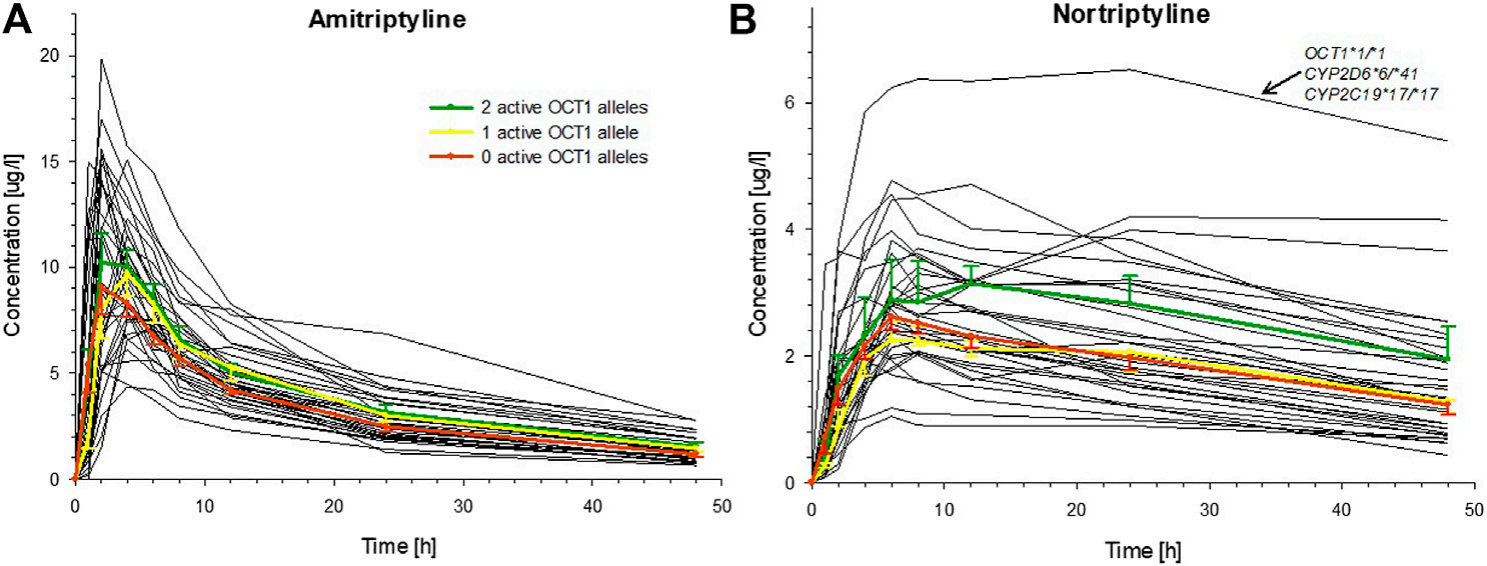
Individual plasma concentrations of **(A)** AT and **(B)** NT over time for all healthy volunteers (black curves), indicating the large interindividual variation for these tricyclic antidepressants. The mean (±SEM) concentrations for carriers of two (green), one (yellow), or zero (red) active OCT1 alleles are superimposed (OCT1-dependent differences in AUC were not statistically significant). The single participant with the highest NT concentrations had wild-type OCT1 genotype, reduced activity CYP2D6 genotype, and a very high activity CYP2C19 genotype.

Large variation was seen in the pharmacokinetics of AT and, even more so, for its therapeutically active metabolite NT. The AUC_inf_ of AT varied about fourfold (range: 109.9–429.9 h*µg/L) and the AUC_48h_ of NT approximately sevenfold (range: 39.3–283.7 h*µg/L). However, these variations were apparently not a result of OCT1 polymorphism, as differences in AUC between carriers of two, one, or zero active OCT1 alleles were not statistically significant (Figure 3; Table 2, Supplementary Figure S1; Supplementary Table S2). The only statistically significant difference in relation to OCT1 genotype was observed for the T_max_ of NT (*p* = 0.016, Jonckheere-Terpstra test), which was almost twofold higher in the group comprised of the carriers of two active OCT1 alleles as compared to the other two groups. However, this difference is likely explained by one subject with particularly high plasma NT concentrations, who had low CYP2D6 activity and very high CYP2C19 activity (Figure 3). Any differences in the AUC_48h_ of the ‘active moiety’ (sum of the AUC_48h_ of AT and NT) between the OCT1 genotypes were not significant (*p* = 0.059, Jonckheere-Terpstra test).

**TABLE 2:** Pharmacokinetic parameters stratified by OCT1 genotype.

Parameter	2 active OCT1 alleles (*n* = 14)	1 active OCT1 allele (*n* = 9)	0 active OCT1 alleles (*n* = 12)	*p*-value^a^
Amitriptyline				
t_1/2_ (h)	21.0 ± 4.0	20.5 ± 3.9	20.3 ± 3.8	0.715
T_max_ (h)	2.9 ± 1.4	3.1 ± 1.0	3.4 ± 1.6	0.725
C_max_ (µg/L)	11.6 ± 4.0	10.5 ± 3.3	9.9 ± 3.9	0.301
AUC_inf_ (h*µg/l)	242.6 ± 87.5	228.5 ± 72.4	199.3 ± 60.0	0.235
AUC_48h_ (h*µg/l)	194.2 ± 66.3	184.2 ± 56.0	161.6 ± 40.5	0.260
CL (L/min)	1.9 ± 0.6	2.0 ± 0.8	2.3 ± 0.6	0.235
V_z_ (L)	3,401 ± 1,041	3,610 ± 1,633	3,828 ± 800	0.260
Nortriptyline				
t_1/2_ (h)	40.1 ± 38.2	56.4 ± 31.3	46.1 ± 24.4	0.742
T_max_ (h)	11.5 ± 8.7	6.7 ± 1.3	6.2 ± 1.4	**0.016**
C_max_ (µg/L)	3.4 ± 1.4	2.6 ± 0.9	2.7 ± 0.7	0.223
AUC_48h_ (h*µg/L)	125.7 ± 63.5	88.3 ± 32.2	90.2 ± 27.1	0.100

Data are shown as the mean.

a Differences were analysed for statistical significance using the Jonckheere-Terpstra test. Significant values are highlighted in bold.

Interestingly, if OCT1*2 would be considered as being fully active, T_max_, C_max_, and AUC_48h_ for NT differed significantly based on OCT1 genotype (*p* = 0.050, 0.018, and 0.011, respectively), whereas any differences in AT pharmacokinetic parameters were still statistically not significant.

**FIGURE 4:**
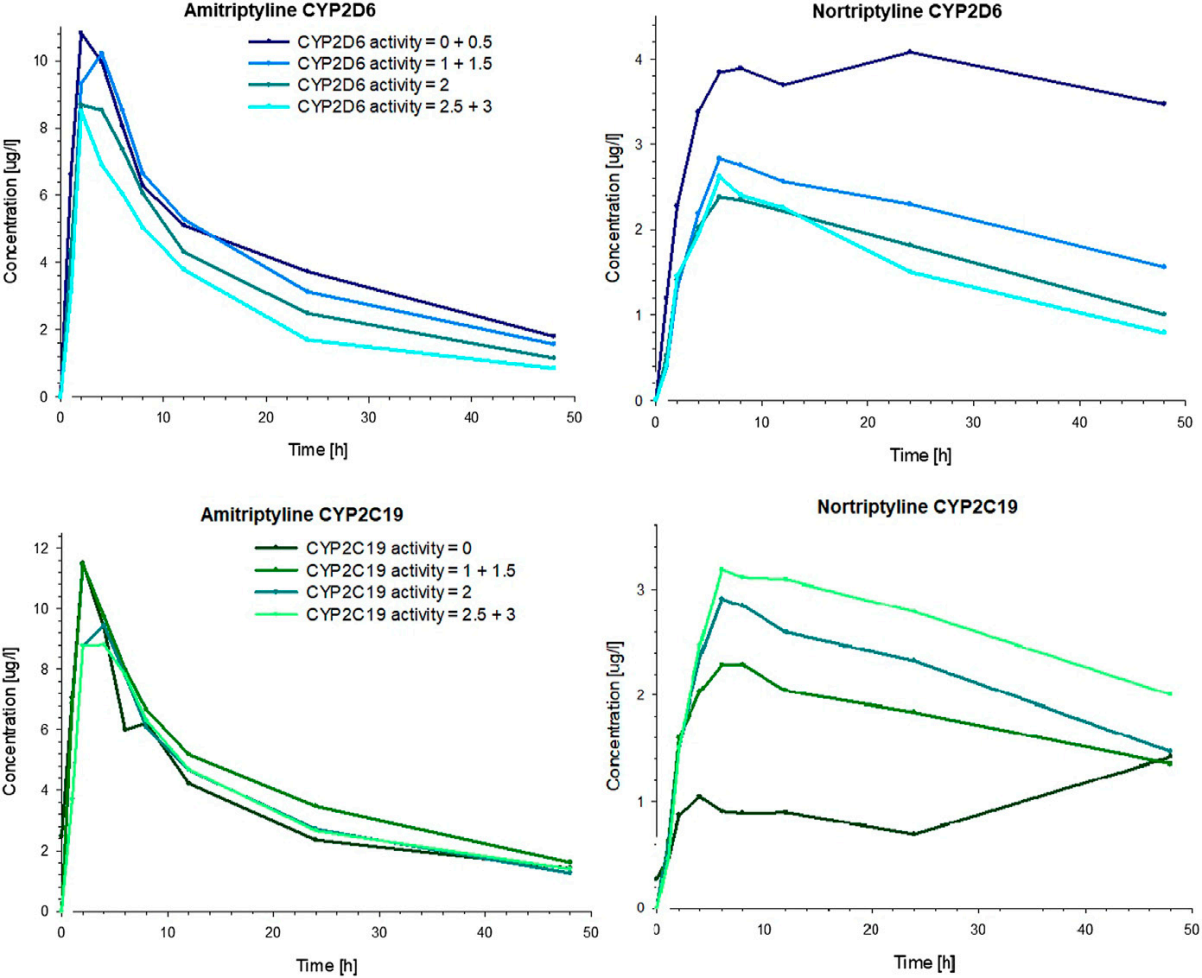
Mean plasma concentrations of AT **(left)** and NT **(right)** in healthy volunteers over time, stratified by the number of active alleles for CYP2D6 **(top)** and CYP2C19 **(bottom)**.

The CYP2D6 genotype had a strong effect on the pharmacokinetics of AT and NT. The plasma concentrations of AT and NT increased with decreasing CYP2D6 activity (Figure 4), and subjects with lower CYP2D6 activity showed a higher AUC_inf_ and AUC_48h_ as well as a longer plasma half-life and a lower AT clearance (Table 3). The CYP2C19 genotype had no significant effect on AT pharmacokinetics (Figure 4) but subjects with higher CYP2C19 activity showed a higher NT AUC_48h_ and C_max_ compared to subjects with lower CYP2C19 activity (Figure 4; Table 4).

**TABLE 3:** Pharmacokinetic parameters stratified by CYP2D6 genotype.

	CYP2D6 activity (semi-quantitative gene dosage)^a^							
**Parameter**	**0** (*n* = 3)	**0.5** (*n* = 2)	**1** (*n* = 10)	**1.5** (*n* = 6)	**2** (*n* = 11)	**2.5** (*n* = 1)	**3** (*n* = 2)	*p*-value**^b^**
Amitriptyline								
t_1/2_ (h)	23.7	24.2	21.4	19.8	19.5	14.0	20.6	**0.046**
T_max_ (h)	3.4	2.0	2.7	3.7	3.4	6.0	2.0	0.677
C_max_ (µg/L)	10.9	12.7	11.9	10.3	10.0	7.0	10.2	0.243
AUC_inf_ (h*µg/L)	310.6	214.4	250.8	225.6	195.5	157.7	157.0	**0.011**
AUC_48h_ (h*µg/L)	236.2	165.9	198.8	183.9	162.2	141.5	130.0	**0.037**
CL (L/min)	1.45	2.09	1.81	2.03	2.25	2.64	2.92	**0.011**
V_z_ (L)	2,982	4,189	3,269	3,446	3,768	3,192	5,361	0.269
Nortriptyline								
t_1/2_ (h)	92.0^c^	105.1	64.2	51.9	29.9^c^	25.0	26.9	**< 0.001**
T_max_ (h)	18.9	15.5	6.2	7.0	7.7	6.0	6.0	0.074
C_max_ (µg/L)	3.48	5.54	2.94	2.79	2.57	3.11	2.49	**0.019**
AUC_48h_ (h*µg/L)	144.6	222.2	103.8	98.3	81.8	89.1	71.1	**0.001**

Data are shown as the mean. The study population was not selected based on their CYP2D6 genotypes.

a The genotype-based CYP2D6 activity is based on the semi-quantitative gene dosage, as described earlier (Steimer et al., 2004).

b Differences were analysed for statistical significance using the Jonckheere-Terpstra test. Significant values are highlighted in bold.

c In 2 subjects carrying zero active CYP2D6 alleles and in one subjects with a CYP2D6 gene activity of 2, no decrease in NT concentration was observed and, therefore, no terminal elimination rate could be calculated.

**TABLE 4:** Pharmacokinetic parameters stratified by CYP2C19 genotype.

CYP2C19 activity score^a^							
**Parameter**	**0** (*n* = 1)	**1** (*n* = 5)	**1.5** (*n* = 2)	**2** (*n* = 16)	**2.5** (*n* = 10)	**3** (*n* = 1)	***p*-value** **^b^**
Amitriptyline							
t_1/2_ (h)	26.7	21.6	20.3	20.1	19.8	28.3	0.359
T_max_ (h)	4.0	2.6	2.0	3.2	3.6	2.0	0.639
C_max_ (µg/L)	11.5	11.1	16.2	10.5	9.5	13.9	0.303
AUC_inf_ (h*µg/L)	320.9	231.0	317.0	210.0	210.7	271.0	0.254
AUC_48h_ (h*µg/L)	229.0	183.2	255.6	171.8	171.3	198.4	0.238
CL (L/min)	1.30	1.94	1.5	2.1	2.2	1.54	0.254
V_z_ (L)	3,004	3,525	2,559	3,696	3,740	3,762	0.340
Nortriptyline^c^							
t_1/2_ (h)	106.3	48.2	25.1	47.7	44.1	145	0.983
T_max_ (h)	6.0	6.0	14.1	6.6	9.9	24.9	0.452
C_max_ (µg/L)	1.05	2.1	3.5	3.0	3.1	6.5	**0.012**
AUC_48h_ (h*µg/L)	39.3	69.4	123	102.8	107.8	283.7	**0.008**

Data are shown as the mean. The study population was not selected based on their CYP2C19 genotypes.

a For calculating the CYP2C19 activity score, CYP2C19*2 was regarded as zero active, CYP2C19*1 was classified as 1, and CYP2C19*17 as 1.5.

b Differences were analysed for statistical significance using the Jonckheere-Terpstra test. Significant values are highlighted in bold.

c In two subjects with CYP2C19*1/*17 genotype and in one subject with CYP2C19*1/*1 genotype, no decrease in NT concentration was observed and, therefore, no terminal elimination rate could be calculated.

A multiple linear regression analysis confirmed statistically significant effects of CYP2D6 genotype on AT pharmacokinetics (Table 5). CYP2D6 genotype accounts for 43% of the variation. Concerning NT, both CYP2D6 and CYP2C19 genotypes had statistically significant effects on the AUC_48h_ and could explain 58% of the variation. In contrast, OCT1 genotype, gender, age, body mass index, and glomerular filtration rate had no significant effects on the variation in both the AUC_inf_ of AT and the AUC_48h_ of nortiptyline.

**TABLE 5:** Multiple linear regression analysis to determine the individual factors that influence AT and NT AUC in healthy volunteers.

	Amitriptyline AUC_inf_ (all factors: *r* = 0.66, *r* ^2^ = 0.43)		Nortriptyline AUC_48h_ (all factors: *r* = 0.76, *r* ^2^ = 0.58)	
Individual factors	Coefficient	*p*-value	Coefficient	*p*-value
Sex	−42.5	0.10	4.58	0.74
Age (years)	−1.13	0.59	−1.39	0.23
Body mass index (kg/m^2^)	1.65	0.78	−4.11	0.21
Glomerular filtration rate (ml/min/1.73 m^2^)	1.02	0.28	−0.02	0.97
OCT1 activity	10.40	0.45	11.56	0.14
CYP2C19 activity	−25.29	0.18	35.94	**0.001**
CYP2D6 activity	−53.07	**0.002**	−30.94	**0.001**

### Adverse Effects of Amitriptyline

**FIGURE 5:**
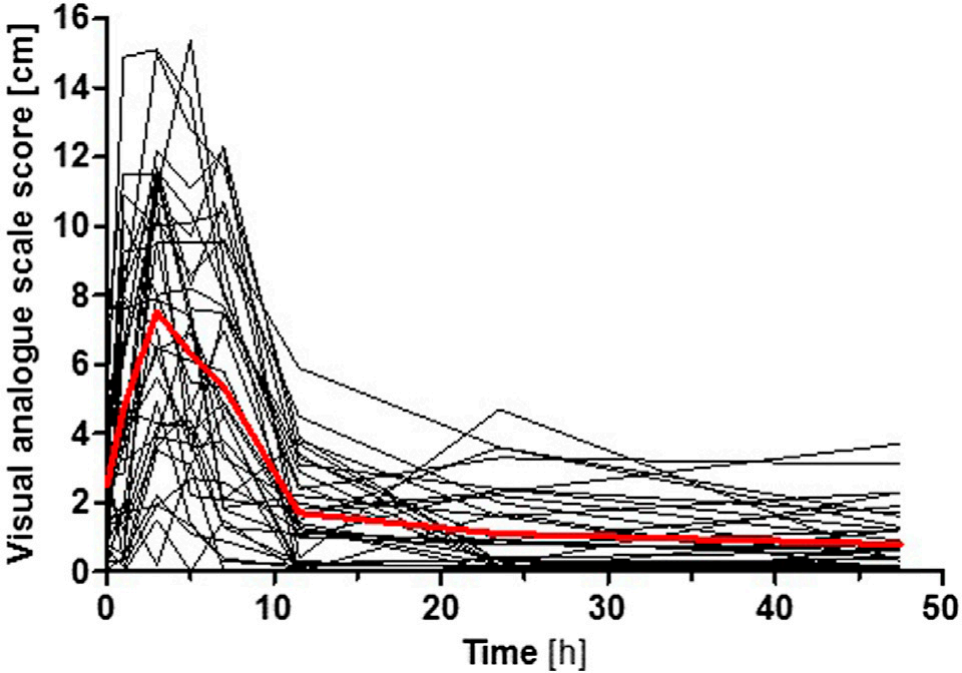
Intensity of fatigue after AT administration reported by the participants using a visual analogue scale. Shown is the time course for each participant (black curves) and the mean superimposed (red curve). The intensity of fatigue was not dependent on OCT1, CYP2D6, or CYP2C19 genotypes (*p* > 0.05, Jonckheere-Terpstra test).

AT was generally well-tolerated and no serious adverse events occurred during the entire study. Using visual analogue scales, the participants reported symptoms of fatigue, which peaked at 3 h after AT administration at which plasma AT concentrations were generally the highest (Figure 5). However, it should be taken into consideration that no placebo control was used in this pharmacokinetic study, and a fully valid assessment of adverse effects was thus not possible (i.e. reported adverse effects might not exclusively be due to AT administration but could be a result of the ‘placebo effect’ as well). The intensity of fatigue was not dependent on OCT1, CYP2D6, or CYP2C19 genotypes (*p* > 0.05, Jonckheere-Terpstra test). Statistically significant time- and concentration-related adverse effects like dry mouth, visual or hearing impairment, restlessness, headache, nausea, dizziness, or a sensation of cold reported using visual analogue scales as well as potential anticholinergic effects studied by pupillometry were not observed after the 25 mg AT dose.

### Pharmacokinetics of Amitriptyline and Nortriptyline in Relation to OCT1 Genotype in Depressive Disorder Patients

**FIGURE 6:**
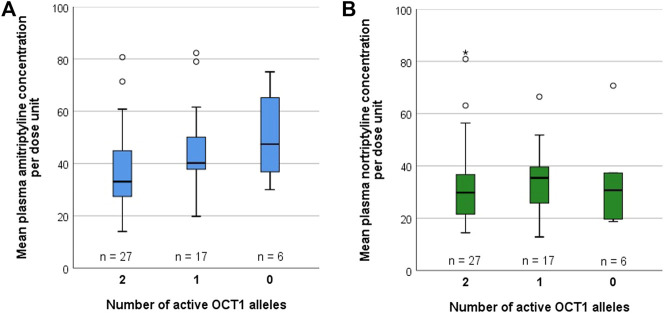
Mean plasma concentrations per dose unit of **(A)** AT and **(B)** NT, stratified by OCT1 genotype, in 50 depressive disorder patients who underwent AT therapy.

Possible differences due to OCT1 polymorphism on the pharmacokinetics of AT and its metabolite NT were additionally studied in 50 patients suffering from medium-grade to severe depressive disorder that were recruited as part of a previous study on the impact of CYP2D6 and CYP2C19 polymorphism on AT and NT pharmacokinetics, adverse effects, and therapy response (Steimer et al., 2004; Steimer et al., 2005). These underwent a therapy of 75 mg AT twice daily at 12 h dosing intervals. Out of these 50 patients, 27 were carriers of two active OCT1 alleles (OCT1*1/*1; Supplementary Table S3), 17 were carriers of one active OCT1 allele (*1 in combination with *2, *3, or *4), and six patients carried zero active OCT1 alleles (*2, *3, or *4). Different CYP2D6 and CYP2C19 genotypes were found to be similarly distributed across all three groups (Table 6). A trend of increasing plasma concentrations with decreasing OCT1 activity was seen for AT (Figure 6, Supplementary Figure S2; Supplementary Table S3). Although the differences in mean AT concentrations between the three groups were rather modest, they showed statistical significance (*p* = 0.018, Jonckheere-Terpstra test). In contrast, mean plasma NT concentrations per dose unit were relatively similar for all OCT1 genotypes. Differences in the ‘active moiety’ (sum of AT and NT plasma concentrations) between OCT1 genotypes were significant (*p* = 0.036, Jonckheere-Terpstra test). Multiple linear regression analysis showed significant effects for OCT1 and CYP2C19 on AT and highly significant effects for CYP2D6 on NT mean plasma concentrations per dose unit (Table 7).

**TABLE 6:** Distribution of CYP2D6 and CYP2C19 activity across the study sample of 50 depressive disorder patients, stratified by OCT1 genotype.

	2 active OCT1 alleles (*n* = 27)	1 active OCT1 allele (*n* = 17)	0 active OCT1 alleles (*n* = 6)
**CYP2D6 activity**			
**3.0**	0 (0%)	1 (6%)	0 (0%)
**2.0**	10 (37%)	8 (47%)	3 (50%)
**1.5**	8 (30%)	2 (12%)	1 (17%)
**1.0**	7 (26%)	5 (29%)	2 (33%)
**0.5**	2 (7%)	1 (6%)	0 (0%)
**CYP2C19 activity**			
**2.0**	16 (59%)	11 (65%)	4 (67%)
**1.0**	10 (37%)	6 (35%)	2 (33%)
**0.0**	1 (4%)	0 (0%)	0 (0%)

**TABLE 7:** Multiple linear regression analysis to determine the individual factors that influence mean plasma AT and NT concentrations per dose unit in depressive disorder patients.

	Mean AT concentration per dose unit (all factors: *r* = 0.45, *r* ^2^ = 0.20)		Mean NT concentration per dose unit (all factors: *r* = 0.68, *r* ^2^ = 0.47)	
Individual factors	Coefficient	*p*-value	Coefficient	*p*-value
OCT1 activity	−7.80	**0.018**	−1.33	0.608
CYP2C19 activity	−10.99	**0.012**	5.96	0.083
CYP2D6 activity	−1.90	0.653	−19.96	**<0.001**

If OCT1*2 would be considered as being fully active, 44 patients would be carriers of two active OCT1 alleles, five patients would be carriers of one active OCT1 allele, and one patient would be a carrier of zero active OCT1 alleles. With this classification, the mean plasma concentrations per dose unit were not significantly different between OCT1 genotypes for both AT (*p* = 0.216, Jonckheere-Terpstra test), and NT (*p* = 0.800, Jonckheere-Terpstra test), but a trend of increasing plasma concentrations with decreasing OCT1 activity was still seen for AT.

### Effects of OCT1 Activity on Plasma Concentrations of Acylcarnitine Derivatives

**FIGURE 7:**
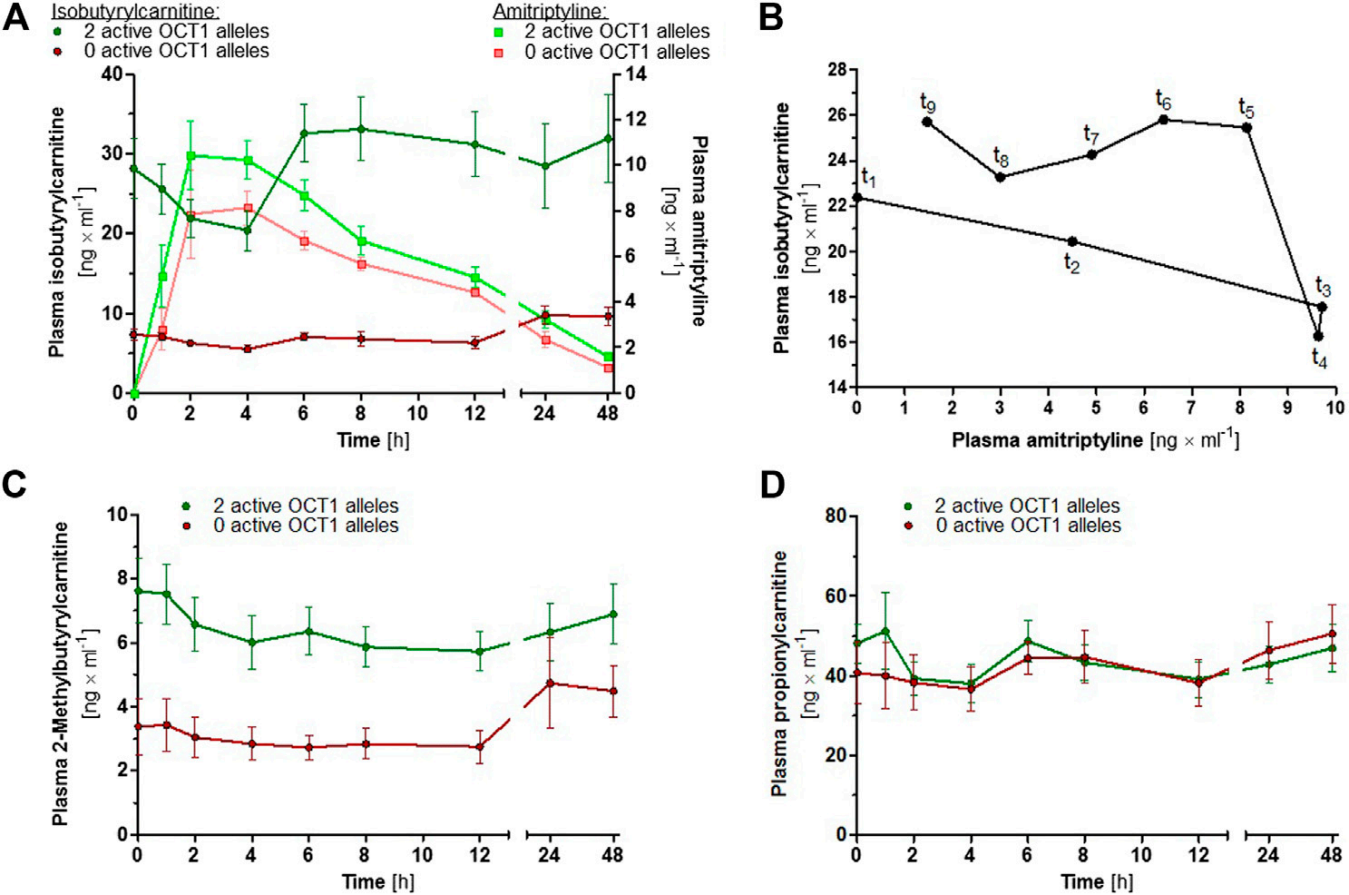
**(A)** Mean ± SEM of IBC (dark coloured circles) and AT (light coloured squares) plasma concentrations over time, stratified by OCT1 genotype (green data points represent two active OCT1 alleles and red data points represent zero active OCT1 alleles; carriers of OCT1*2 were not included). **(B)** Hysteresis plot showing the mean plasma concentrations of AT and IBC in 13 healthy volunteers with two active OCT1 alleles. **(C)** Mean ± SEM of 2-methylbutyrylcarnitine and **(D)** propionylcarnitine plasma concentrations over time, stratified by OCT1 genotype.

In order to investigate the proposed suitability of IBC as a human *in vivo* biomarker for OCT1 activity (Luo et al., 2020), plasma concentrations of IBC as well as of 2-methylbutyrylcarnitine and propionylcarnitine were determined in a subgroup of 18 volunteers who participated in the study on AT pharmacokinetics. Because of the ambiguous role of OCT1*2 with respect to several OCT1 substrates, carriers of OCT1*2 were not included. Baseline IBC plasma concentrations were 2.9- to 4.9-fold and 2-methylbutyrylcarnitine plasma concentrations were 1.3- to 2.3-fold higher in participants with two active OCT1 alleles compared to the participants with zero active OCT1 alleles (*p* < 0.0001 for both, unpaired *t* test; Figures 7A,C), whereas plasma propionylcarnitine concentrations were similar for both groups (Figure 7D; *p* = 0.386, unpaired *t* test). At time points 2 and 4 h after AT administration, at which plasma AT concentrations were generally the highest, plasma IBC concentrations were reduced to 72 and 67% of the baseline IBC concentrations (*p* = 0.001 and 0.002, paired *t* test; Figure 7A).

## Discussion

In this study, the effects of OCT1 polymorphism on AT and NT pharmacokinetics were investigated comprehensively in healthy volunteers and in depressive disorder patients. With their relatively high pK_a_ values, most tricyclic antidepressants could be typical OCT1 substrates, and this hypothesis was further supported by the fact that all tested tricyclic antidepressants were moderate to strong inhibitors of OCT1 (Figure 2). Yet, in our two studies in healthy volunteers and patients, there was no strong and consistent effect of OCT1 on the pharmacokinetics of AT and its active metabolite NT. This indicates that non-ionic diffusion, independent of transporter activity, likely is the main mechanism of biological membrane passage or, alternatively, other transporters are involved. Transporter-mediated hepatocyte uptake could be demonstrated with saturable transport kinetics for imipramine (Hallifax and Houston, 2007), another tricyclic antidepressant with similar lipophilicity. Possible candidates might include the OCTN1 and OCTN2 transporters as well as the proton-coupled organic cation antiporter that has been described in the literature but has not yet been identified on the molecular level (Tega et al., 2021).

While no statistically significant effects for OCT1 polymorphism were observed in healthy volunteers, a moderate trend of increasing plasma concentrations with decreasing OCT1 activity was seen for AT in depressive disorder patients. A possible reason for this discrepancy might be the differences in dose and duration. While the healthy volunteers were given a single dose of 25 mg of AT, the depressive disorder patients received a total of 150 mg per day and measurements were taken over two weeks after steady-state has been achieved. With regard to NT pharmacokinetics, both studies were concordant in that OCT1 does not appear to be a major determining factor.

The fact that only a single dose of AT was given in the study in healthy volunteers and that, accordingly, steady-state plasma concentrations were not achieved, is a potential limitation of this study. Also, it cannot be excluded that OCT1 effects might still be observed at higher dosage. The average C_max_ for AT in the healthy volunteers was 10.7 µg/L, or 0.039 µM, which is 730-fold lower than the IC_50_ of 28.6 µM determined in our *in vitro* assays.

**FIGURE 8:**
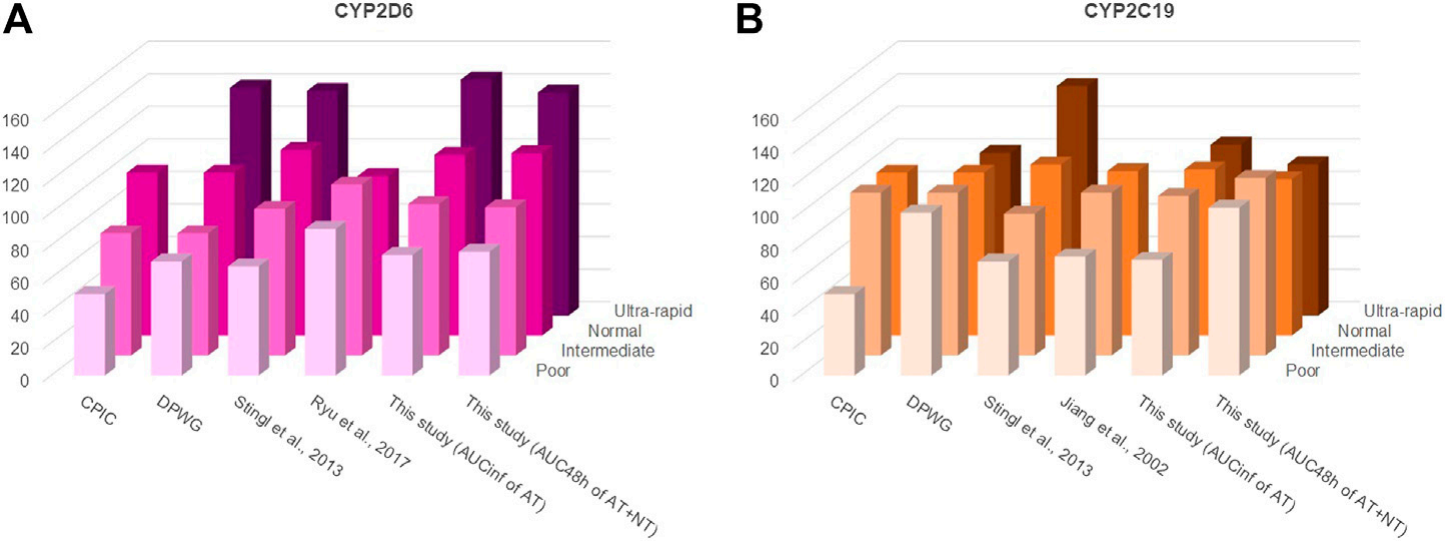
Different starting dosage adjustment recommendations from the literature and based on the results of this study for **(A)** CYP2D6 and **(B)** CYP2C19. These were taken from the Clinical Pharmacogenetics Implementation Consortium (CPIC^®^) guideline (Hicks et al., 2017), the Dutch Pharmacogenetics Working Group (DPWG) guideline (August 2019 update), or calculated based on the formulas described by Stingl et al. (2013) and by using the AUCs determined in the respective studies. In accordance with the CPIC^®^ and DPWG final consensus on CYP2D6 genotype to phenotype (Caudle et al., 2020), a CYP2D6 activity score of 0 was classified in this study as poor, of 0.5 and 1 as intermediate, of 1.5 and 2.0 as normal/extensive, and of >2.5 as ultra-rapid metaboliser phenotypes. For CYP2C19, an activity score of 0 was classified in this study as poor, of 1 as intermediate, of 1.5 and 2 as normal/extensive, and of >2 as ultra-rapid metaboliser phenotypes. The starting dosage adjustment recommendations are also listed in Supplementary Table S4. As apparent, there is a high consistency between different recommendations and the measurements from this study, particularly with regard to the CYP2D6 genotype.

While it is apparently not necessary to take the OCT1 genotype into consideration for AT or NT dosing, CYP2C19 and CYP2D6 genotypes are highly relevant and AT or NT dosage should be adjusted accordingly (Brockmöller et al., 2000; Hicks et al., 2017). Several approaches have been proposed by different groups but their suggestions are essentially in concordance. Figure 8 and Supplementary Table S4 show earlier recommendations on CYP2D6 and CYP2C19 genotype-based dose adjustments by the Clinical Pharmacogenetics Implementation Consortium (CPIC®; Hicks et al., 2017), the Dutch Pharmacogenetics Working Group (DPWG; guidelines update August 2019), and based on the pharmacokinetic data from more recent clinical studies and from this study by using the calculations described in Stingl et al. (2013). The dosage adjustment recommendations based on the data from this study were similar to those calculated previously by Stingl et al. (2013), except when using the sum of the AUC_48 h_ of AT and NT for calculating adjustments for CYP2C19 poor and ultra-rapid metabolisers. This is likely due to the strong impact this enzyme has on the NT pharmacokinetics.

OCT1 is able to transport a large number of different compounds, among them many drugs, but its physiological function is not yet understood. As some endogenous acylcarnitines were shown to be OCT1 substrates, a potential physiological role of OCT1 could be the regulation of intracellular concentrations of these carnitine derivatives. It has been proposed that IBC could serve as an endogenous biomarker (Luo et al., 2020), which might be useful for further studying OCT1 activity in humans. Our results confirm its suitability, as up to five-fold differences in IBC plasma concentrations between participants with normal OCT1 activity and carriers of zero active OCT1 alleles were observed. Moreover, peak plasma concentrations of the OCT1 inhibitor AT correlated with a transient reduction in plasma IBC concentrations (Figure 7). The average peak plasma concentration of AT was 10.8 μg/L. With 95% plasma protein binding (Hardman et al., 2001), the peak concentration of unbound AT was 0.54 μg/L. Based on the calculations by Ahlin et al. and Ito et al. (Ito et al., 1998; Ito et al., 2002; Ahlin et al., 2008), the maximum concentration of unbound AT in the portal vein was estimated to be 745.9 µg/L or 2.69 µM. At this concentration, 23% OCT1 inhibition was achieved *in vitro*, which corresponds to the 33% decrease in IBC plasma concentration observed *in vivo*. Here, it can be concluded that IBC might indeed be a suitable endogenous OCT1 biomarker. 2-Methylbutyrylcarnitine could be considered as well, as OCT1-dependent differences were also observed, although these were less pronounced and plasma concentrations were generally lower than those of IBC. Despite the structural similarity, propionylcarnitine plasma concentrations were not affected by OCT1 genetic variation. A speculative but possible explanation for the reduction in plasma IBC concentrations at these time points could be a potential inhibition of OCT1 by high plasma AT concentrations. Alternatively, this association could be the result of complex metabolic crosstalk. As a placebo control was not part of this mainly pharmacokinetic study, possible effects due to diurnal rhythm and nutrition cannot be excluded.

In conclusion, the pharmacokinetics of AT and NT are strongly dependent on the CYP2C19 and CYP2D6 genotypes, while OCT1 polymorphism does not appear to be a major medically relevant factor. It thus remains to be elucidated which organic cation transporter(s) are relevant for intestinal absorption, hepatic uptake, and passage across the blood-brain barrier.

## Data Availability

The raw data supporting the conclusions of this article will be made available by the authors, without undue reservation.

## Abbreviations

ASP^+^, 4-(4-(dimethylamino)styryl)-*N*-methylpyridinium iodide; AT, amitriptyline; AUC, area under the curve; CYP, cytochrome P450; IBC, isobutyrylcarnitine; NT, nortriptyline; OCT, organic cation transporter; SLC, solute carrier.
