# Supplementary material for: Effects of Genetic Polymorphism in CYP2D6, CYP2C19, and the Organic Cation Transporter OCT1 on Amitriptyline Pharmacokinetics in Healthy Volunteers and Depressive Disorder Patients
Source: Front Pharmacol. 2021 May 21;12:688950. doi: 10.3389/fphar.2021.688950 (PMC8175851; doi:10.3389/fphar.2021.688950)
Supplement: Supplementary file 1 [file DataSheet1.PDF]

Table S1 Mass spectrometry detection parameters of analytes and internal standards

**Compound**	**Retention time** (min)	**Mass Q1** (Da)	**Mass Q3** (Da)	**Declustering Potential** (V)	**Collision Energy** (V)	**Collision cell exit potential** (V)
Amitriptyline	11	278.2	91.0	36	36	16
Amitriptyline-d6	9	284.3	91.0	71	34	7
Nortriptyline	17	264.2	233.2	46	21	16
Nortriptyline-d3	15	267.4	91.0	71	30	7
Isobutyrylcarnitine	4	232.1	85.0	70	25	15
Isobutyrylcarnitine-d6	4	238.1	85.0	70	25	15
2-Methylbutyrylcarnitine	6	246.2	85.0	71	29	4
2-Methylbutyrylcarnitine-d9	6	255.4	85.0	71	29	4
Propionylcarnitine	4	218.2	85.0	60	28	6
Propionylcarnitine-d3	4	221.2	85.0	60	28	6
Propionylcarnitine-d9	4	227.6	85.0	60	28	6

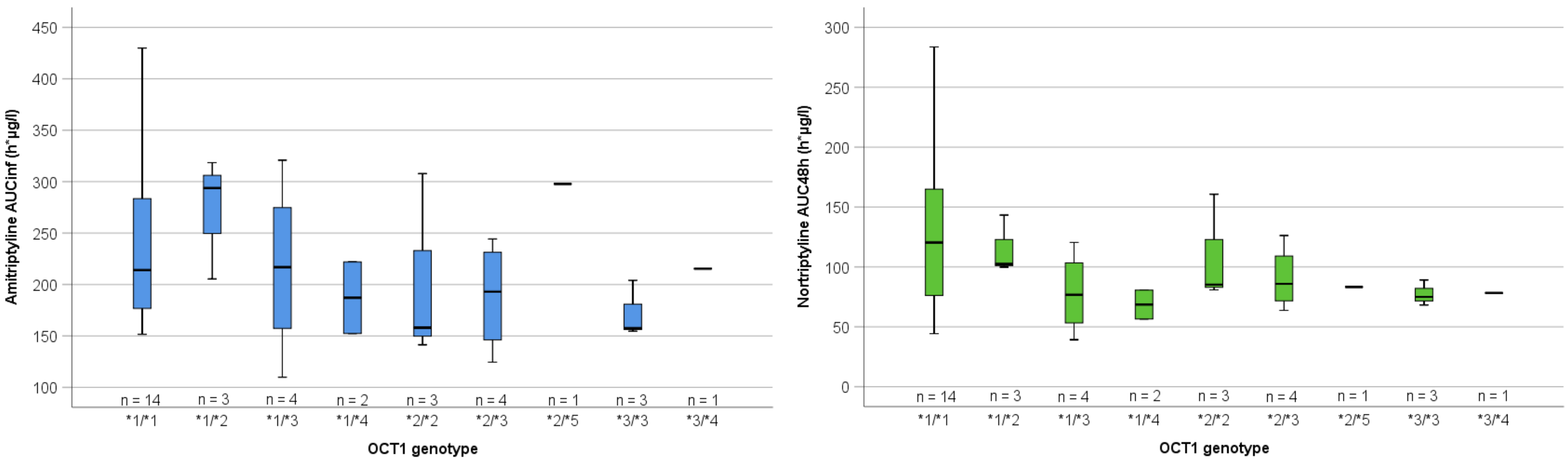

Figure S1 (**A**) Amitriptyline AUC_{inf} and (**B**) Nortriptyline AUC_{48h} in 35 healthy volunteers, stratified by OCT1 genotype

Table S2 Mean ± standard deviation of amitriptyline and nortriptyline AUC in 35 healthy volunteers, stratified by OCT1 genotype

**OCT1 genotype**	**Number of study participants**	**Amitriptyline AUC_{inf}** (h*µg/l)	**Nortriptyline AUC_{48h}** (h*µg/l)
*1 / *1	14	243 ± 88	126 ± 63
*1 / *2	3	273 ± 59	115 ± 24
*1 / *3	4	216 ± 87	78 ± 34
*1 / *4	2	187 ± 49	69 ± 17
*2 / *2	3	202 ± 92	109 ± 45
*2 / *3	4	189 ± 53	90 ± 27
*2 / *5	1	298	83
*3 / *3	3	172 ± 28	77 ± 11
*3 / *4	1	215	78

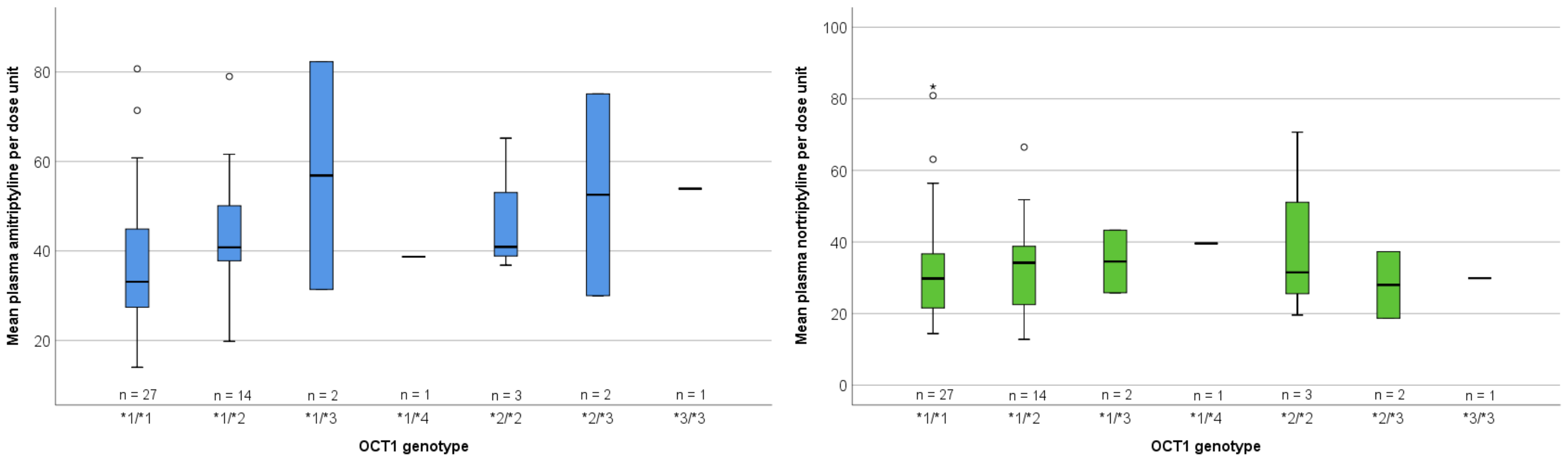

Figure S2 Mean plasma concentrations per dose unit for (**A**) amitriptyline and (**B**) nortriptyline in 50 patients, stratified by OCT1 genotype

Table S3 Mean ± standard deviation of amitriptyline and nortriptyline plasma concentrations per dose unit in 50 patients, stratified by OCT1 genotype

OCT1 genotype	Number of study participants	Mean plasma concentration per dose unit	
		Amitriptyline	Nortriptyline
*1 / *1	27	37.3 ± 15.9	34.0 ± 18.3
*1 / *2	14	44.0 ± 15.2	33.5 ± 14.6
*1 / *3	2	56.9 ± 36.0	34.6 ± 12.4
*1 / *4	1	38.7	39.6
*2 / *2	3	47.6 ± 15.4	40.6 ± 26.7
*2 / *3	2	52.6 ± 31.9	28.0 ± 13.2
*3 / *3	1	53.9	29.9

Table S4 Different starting dosage adjustment recommendations from the literature and based on the results of this study

Metaboliser phenotype	CPIC®[a]	DPWG[b]	Stingl et al., 2013	Jiang et al., 2002[c]	Ryu et al., 2017[c]	This study[c,d] (AUC_{inf} of AT)	This study[c,d] (AUC_{48h} of AT+NT)
**CYP2D6**							
Poor	50 %	70 %	67 %		90 %	74 %	76 %
Intermediate	75 %	75 %	90 %		105 %	93 %	91 %
Normal	100 %	100 %	114 %		98 %	111 %	112 %
Ultra-rapid		140 %	138 %			145 %	137 %
**CYP2C19**							
Poor	50 %	100 %	70 %	73 %		71 %	103 %
Intermediate	100 %	100 %	87 %	100 %		98 %	109 %
Normal	100 %	100 %	105 %	101 %		102 %	96 %
Ultra-rapid		100 %	141 %			105 %	93 %

[a]CPIC®, Clinical Pharmacogenetics Implementation Consortium guideline (Hicks et al., 2017)
[b]DPWG, Dutch Pharmacogenetics Working Group guideline (August 2019 update)
[c]Dose adjustment recommendations were calculated based on the formulas described by Stingl et al., 2013 and by using the AUCs determined in the respective studies
[d]In accordance with the CPIC® and DPWG final consensus on CYP2D6 genotype to phenotype (Caudle et al., 2020), a CYP2D6 activity score of 0 was classified as poor, of 0.5 and 1 as intermediate, of 1.5 and 2.0 as normal/extensive, and of >2.5 as ultra-rapid metaboliser phenotypes. For CYP2C19, an activity score of 0 was classified as poor, of 1 as intermediate, of 1.5 and 2 as normal/extensive, and of >2 as ultra-rapid metaboliser phenotypes.
